# Treatment planning of VMAT and step‐and‐shoot IMRT delivery techniques for single fraction spine SBRT: An intercomparative dosimetric analysis and phantom‐based quality assurance measurements

**DOI:** 10.1002/acm2.12788

**Published:** 2019-12-10

**Authors:** Zi Ouyang, Danielle V. LaHurd, Ehsan H. Balagamwala, Samuel T. Chao, John H. Suh, Ping Xia

**Affiliations:** ^1^ Department of Radiation Oncology Taussig Cancer Institute Cleveland Clinic Cleveland OH USA

**Keywords:** IMRT, quality assurance, spine SBRT, treatment planning, VMAT

## Abstract

**Purpose:**

To retrospectively compare clinically treated step‐and‐shoot intensity modulated radiotherapy (ssIMRT) and volumetric modulated arc therapy (VMAT) spine stereotactic body radiotherapy (SBRT) plans in dosimetric endpoints and pretreatment quality assurance (QA) measurements.

**Methods:**

Five single fraction spine SBRT (18 Gy) cases — including one cervical, two thoracic, and two lumbar spines — clinically treated with ssIMRT were replanned with VMAT, and all plans were delivered to a phantom for comparing plan quality and delivery accuracy. Furthermore, we analyzed 98 clinically treated plans (18 Gy single fraction), including 34 ssIMRT and 29 VMAT for cervical/thoracic spine, and 19 ssIMRT and 16 VMAT for lumbar spine. The conformality index (CI) and homogeneity index (HI) were calculated, and QA measurement records were compared. For the spinal cord/cauda equina, the maximum dose to 0.03 cc (D_0.03cc_) and volume receiving 10 or 12 Gy (V_10Gy_/V_12Gy_) were recorded. Statistical significance was tested with the Mann–Whitney U test.

**Results:**

Compared to ssIMRT, replanned VMAT plans had lower V_10Gy_/V_12Gy_ and D_0.03cc_ to the spinal cord/cauda equina in all five cases, and better CI in three out of five cases. The VMAT replans were slightly less homogeneous than those of ssIMRT plans. Both modalities passed IMRT QA with >95% passing rate with (3%, 3 mm) gamma criteria. With the 98 clinical cases, for cervical/thoracic ssIMRT and VMAT plans, the median V_10Gy_ of spinal cord was 4.15% and 1.85% (*P* = 0.004); the median D_0.03cc_ of spinal cord was 10.85 Gy and 10.10 Gy (*P* = 0.032); the median CI was 1.28 and 1.08 (*P* = 0.009); the median HI were 1.34 and 1.33 (*P* = 0.697), respectively. For lumbar spine, no significant dosimetric endpoint differences were observed. The two modalities were comparable in delivery accuracy.

**Conclusion:**

From our clinically treated plans, we found that VMAT plans provided better dosimetric quality and comparable delivery accuracy when compared to ssIMRT for single fraction spine SBRT.

## 1 INTRODUCTION

Recent studies^1^, ^2^, ^3^, ^4^, ^5^ report that fast pain relief, excellent local control, and low toxicity are achievable with stereotactic body radiotherapy (SBRT) for the treatment of spinal metastatic diseases. The much larger biological effective dose of SBRT compared to that of conventional radiotherapy (RT) is more effective in overcoming radioresistance.^6^ A complete course of SBRT often consists one to five fractions with 8 to 30 Gy per fraction.^7^ This treatment is made possible with modern radiotherapy technology including inverse planning and optimization algorithms, patient specific quality assurance (QA), image guidance, high definition multi‐leaf collimator (MLC), as well as advanced immobilization. When delivering SBRT on a linear accelerator (Linac) equipped with MLCs, two modalities are often used — step‐and‐shoot intensity modulated radiotherapy (ssIMRT) and volumetric modulated arc therapy (VMAT).

Early studies^8^, ^9^, ^10^ demonstrated the feasibility and benefits of using VMAT in conventionally fractionated radiation treatment. It has also been shown that VMAT reduced the treatment time for different sites including spine and lung SBRT, pediatric pelvic irradiation, and whole‐abdominopelvic irradiation in an early case study.^11^ Especially with the use of flattening filter‐free (FFF) beams, increased dose rate further shortens SBRT treatment time.^12^ Although VMAT has faster and easier delivery compared to ssIMRT, some concerns exist for the use of VMAT in spine SBRT cases where a steep dose fall‐off is required between the boundary of the spinal cord and the tumor. Using the Eclipse treatment planning system in a study with ten patients treated with single fraction spine SBRT,^13^ Wu et al. reported that single‐arc VMAT provided less cord sparing compared to ssIMRT, while two‐arc VMAT was only comparable to ssIMRT. In a study published by Huang et al.,^14^ it was found that VMAT plans had worse conformality than ssIMRT plans while the average D_max_ of the spinal cord was not significantly different between VMAT plans (12.9 ± 1.3 Gy) and ssIMRT plans (12.5 ± 1.3 Gy). In their study, however, the VMAT plans were created using an early version of Pinnacle 9.0 (Philips) while ssIMRT plans were created using iPlan 4.5 (Brainlab). Delivering all 10 plans in the phantom with Novalis TX machine (Varian), it was found that the Gamma indices for IMRT plans were worse than VMAT plans, 98.86% vs. 92.60% (3%, 3 mm) and 92.30% vs. 82.27% (2%, 2 mm). In our institution, we started our spine SBRT program in 2005 with the step‐and‐shoot delivery technique under a team consisting of the same radiation oncologists and neurosurgeons. From 2014 to 2016, we gradually switched our delivery technique from ssIMRT to VMAT. For this study, all treatment plans were created using Pinnacle (V9.6 and V9.10) and treated with Varian Edge with 120HD MLC.

When comparing ssIMRT and VMAT for spine SBRT, previous studies have used research replans.^13^, ^14^, ^15^, ^16^, ^17^ While it is important to keep the same anatomy, research replans are different from clinical plans; research replans are not planned under the same clinical stress (limited time/resources) and do not undergo the scrutiny of treating radiation oncologists. Furthermore, comparison of clinical plans with research plans did not represent clinical practice pattern. To compare ssIMRT and VMAT, beside research replans, we sought to evaluate spine SBRT plans that were clinically planned and delivered.

## 2 MATERIALS AND METHODS

### 2.1 Treatment planning

Radiation Therapy Oncology Group (RTOG) 0631^18^ and institutional guidance were used as acceptance criteria for 18 Gy single fraction spine SBRT plans. Specifically, 90% of the target volume was required to receive 100% of prescription dose. Dose limits to organs at risk (OAR), including the spinal cord, cauda equina, esophagus, and kidneys, are listed in Table 1. The target and spinal cord/cauda equina volumes were contoured on fused CT and MR images, where the spinal cord or cauda equina was contoured starting 3 mm above the superior extent of the target volume to 3 mm below the inferior extent of the target volume, with a 1.5 mm CT slice thickness. All treatment was planned with Pinnacle3 treatment planning systems (TPS) version 9.6 or 9.10 (Philips Radiation Oncology Systems, Fitchburg, WI) for Varian Edge machines (Varian Medical Systems, Palo Alto, CA) with 6 MV FFF beams and 120HD MLC.

**Table 1:** Plan acceptance criteria for target and organs at risks.

ROI	Acceptance criteria
Tumor	V_18Gy_> 90%
Spinal cord	V_10Gy_ < 10%
Spinal cord	D_0.03cc_ < 14 Gy
Cauda equina	V_10Gy_ < 12 Gy
Cauda equina	D_0.03cc_ < 16 Gy
Esophagus	D_2.5cc_ < 14 Gy
Esophagus	D_0.03cc_ < 22 Gy
Whole kidney	V_4Gy_ < 50%

Abbreviation: ROI, region of interest.

### 2.2 Record review

Plan set‐up, patient anatomy, target volume, target‐OAR relationships, and clinical measurements were reviewed on plan and treatment records. A scoring method was used to evaluate the plan complexity: (a) Four scoring elements were defined: vertebral body, left transverse process and articular process, right transverse process and articular process, spinous process; (b) The involvement of each scoring element in the target volume adds one point to the plan complexity score.

### 2.3 Dosimetric analysis

Treatment plans were transferred from the TPS to MIM (MIM Software Inc., Cleveland, OH) for dosimetric analysis. Dose volume histograms (DVHs) for the target and spinal cord/cauda equina were extracted. For the spinal cord and cauda equina, the maximum dose to 0.03 cc (D_0.03cc_) and volume receiving 10 or 12 Gy (V_10Gy_ or V_12Gy_) were recorded. The plan conformality index (CI) and homogeneity index (HI) were calculated using the following equations,

(1) $$\text{CI}=\frac{V_{\text{Rx}}}{V_{\text{target}}},$$

and

(2) $$\text{HI}=\frac{D_{\max}}{D_{\text{Rx}}}.$$

here, V_Rx_ is the volume that received the prescription dose, V_target_ is the volume of the target, D_max_ is the maximum dose, and D_Rx_ is the prescription dose. Phantom QA records were reviewed for each plan, and gamma passing rates (gPR) were used for evaluating delivery accuracy. Plan quality was evaluated using the parameters including V_10Gy_/V_12Gy_ and D_0.03cc_ for spinal cord or cauda equina, CI, and HI. gPR was used to determine plan delivery accuracy. The gamma criteria was (3%, 3mm), and gamma < 1 was considered passing. gPR was the percentage of passing pixels. Statistical significance was tested using two‐sided Mann–Whitney *U* test when applicable, and *P* < 0.05 was considered significant.^19^

### 2.4 Replan‐based comparison

Five ssIMRT plans (Table 2) with cervical (C), thoracic (T), and lumbar (L) spine lesions were selected from an Institutional Review Board (IRB) approved registry to be replanned with VMAT for head‐to‐head comparison. The five ssIMRT and five VMAT plans were measured using the electronic portal imaging device (EPID). EPID IMRT measurement results were analyzed using PerFraction^TM^ (Sun Nuclear Corp., Melbourne, FL).

**Table 2:** Treatment locations and volumes for five step‐and‐shoot intensity modulated radiotheraphy plans selected for volumetric modulated arc theraphy replan.

Plan	Treatment location	Tumor volume (cc)	Complexity score
1	C2	29.78	4
2	T4–6	72.74	2
3	T8–11	280.03	4
4	L3–5	198.19	3
5	L2–4	266.11	3

### 2.5 Population‐based comparison

Seventy four patients treated during 2014–2016 with 18 Gy single fraction spine SBRT for metastases were selected from the IRB approved registry. Tumor location ranged from C1 to L5 vertebral levels. Multi‐isocenter plans were analyzed as individual plans per isocenter, which resulted in a total of 98 plans, which included 34 ssIMRT and 29 VMAT for C/T spine, and 19 ssIMRT and 16 VMAT for L spine. C/T and L spine plans were analyzed separately as the dose constraints to the spinal cord and cauda equina were different. Quality assurance was performed with IBA MatriXX (IBA dosimetry, Bartlett, TN).

## 3 RESULTS

### 3.1 Replan‐based comparison

**Figure 1:**
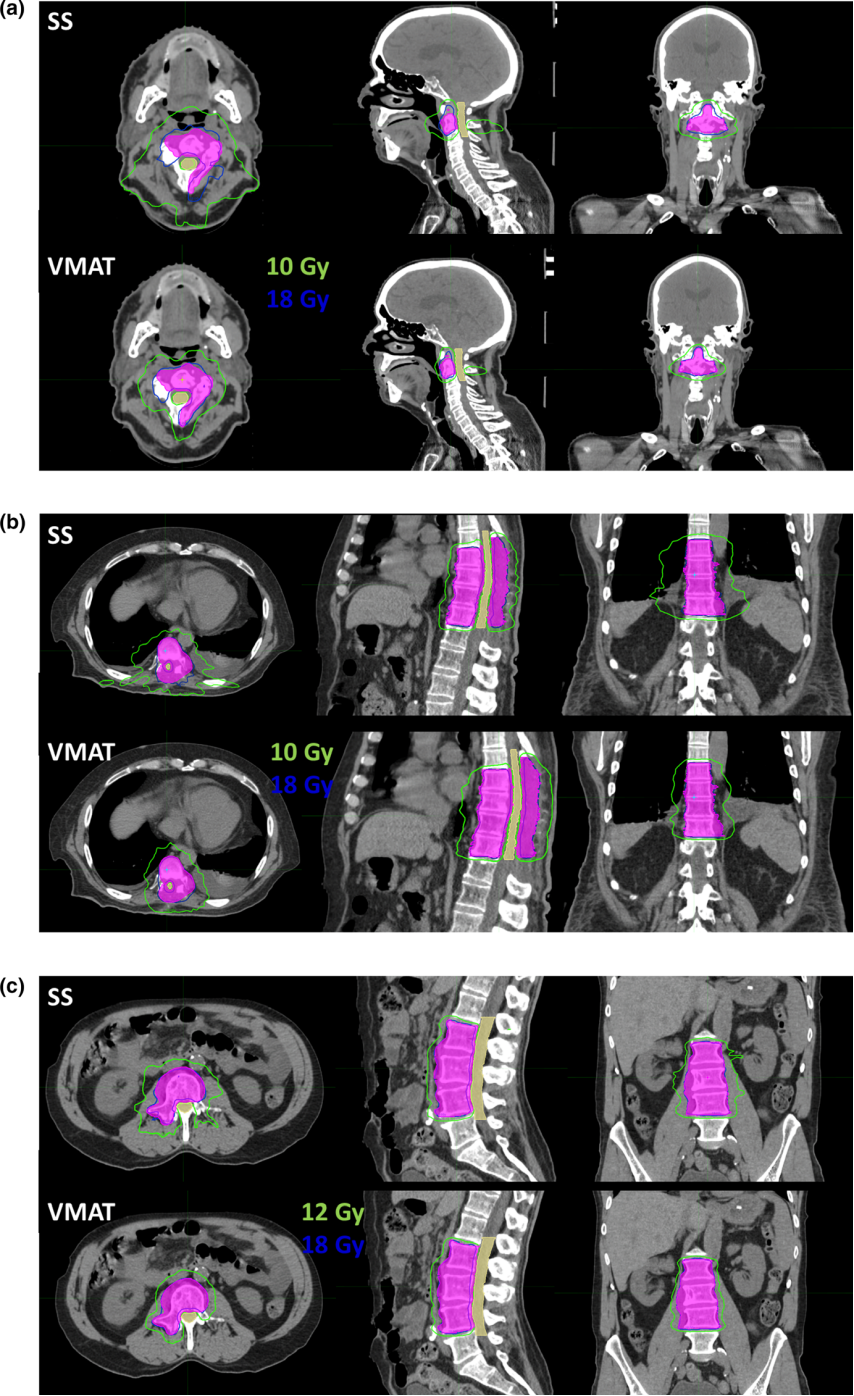
Dose distributions of (a) case 1, C2; (b) case3, T8‐11; and (c) case 5, L2‐4 with step‐and‐shoot intensity modulated radiotheraphy (top) and volumetric modulated arc theraphy (bottom). The tumor and spinal cord are in color wash, and the 18 Gy (blue) and 10 Gy/12 Gy (green) isodose lines are shown.

**Figure 2:**
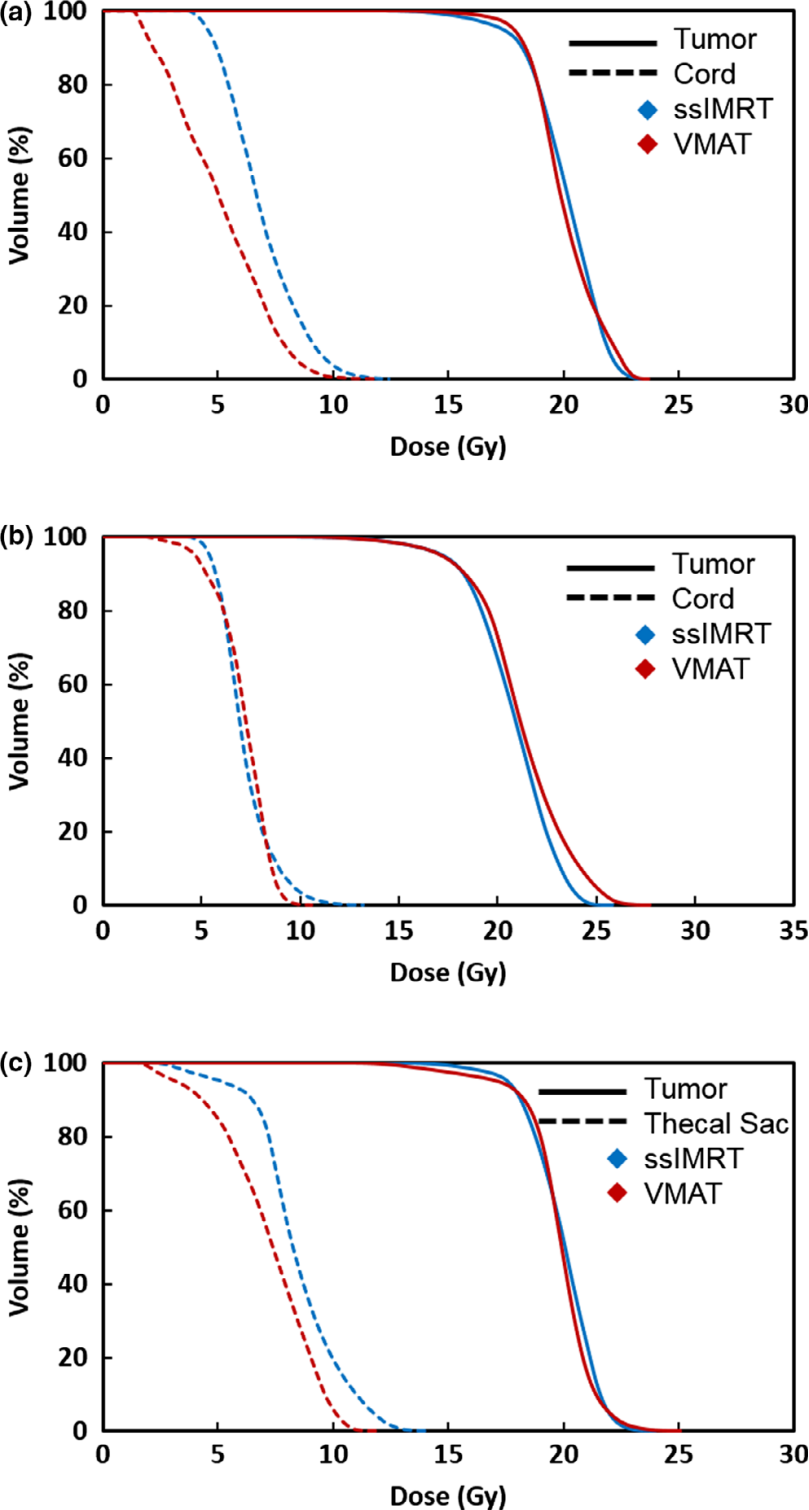
Dose volume histograms of (a) case 1, C2; (b) case3, T8‐11; and (c) case 5, L2‐4 with step‐and‐shoot intensity modulated radiotheraphy (blue) and volumetric modulated arc theraphy (red). The tumor is shown in solid line and the spinal cord is shown in dashed line.

Five clinically treated ssIMRT plans were replanned with VMAT. As shown in Table 2, the treatment locations included C, T, and L spines; the target volumes ranged from 29.78 to 280.03 cc; the complexity scores ranged from 2 to 4 for the five cases. Plan 1, 2, 3, and 5 had nine beams and plan 4 had 6 beams in the clinical ssIMRT plans. For the VMAT replans, plan 1 had one arc, and others had two arcs (Table 3). Both the original and replans met the planning goals. For all five cases, VMAT plans lowered the dose to spinal cord or cauda equina. Figures 1 and 2 show the dose distributions and DVHs of three of five comparison cases with both ssIMRT and VMAT. IMRT QA was performed using EPID with three‐dimensional composite gamma, and all plans had >95% gPR with (3%, 3 mm) gamma criteria.

**Table 3:** Quality of volumetric modulated arc theraphy (VMAT) replans compared to the original step‐and‐shoot intensity modulated radiotheraphy (SSIMRT) plans.

Plan	Beam #		V_10Gy_ (%)		V_12Gy_ (%)		D_0.03cc_ (Gy)		CI		HI		gPR (%)	
	ssIMRT	VMAT	ssIMRT	VMAT	ssIMRT	VMAT	ssIMRT	VMAT	ssIMRT	VMAT	ssIMRT	VMAT	ssIMRT	VMAT
1	9	1	3.59	0.47	0.08	0	11.40	10.00	1.43	1.13	1.30	1.32	98.07	98.23
2	9	2	4.85	0.38	0.02	0	10.70	9.60	1.28	1.17	1.58	1.59	99.74	98.25
3	9	2	3.47	0.14	0.23	0	11.70	9.70	1.12	1.05	1.42	1.54	99.27	98.41
4	6	2	5.04	2.02	0.50	0.05	12.60	11.40	0.97	1.08	1.43	1.49	99.28	98.48
5	9	2	19.40	5.73	3.52	0	13.50	11.20	0.98	0.97	1.33	1.39	99.28	98.07

### 3.2 Population‐based comparison

**Figure 3:**
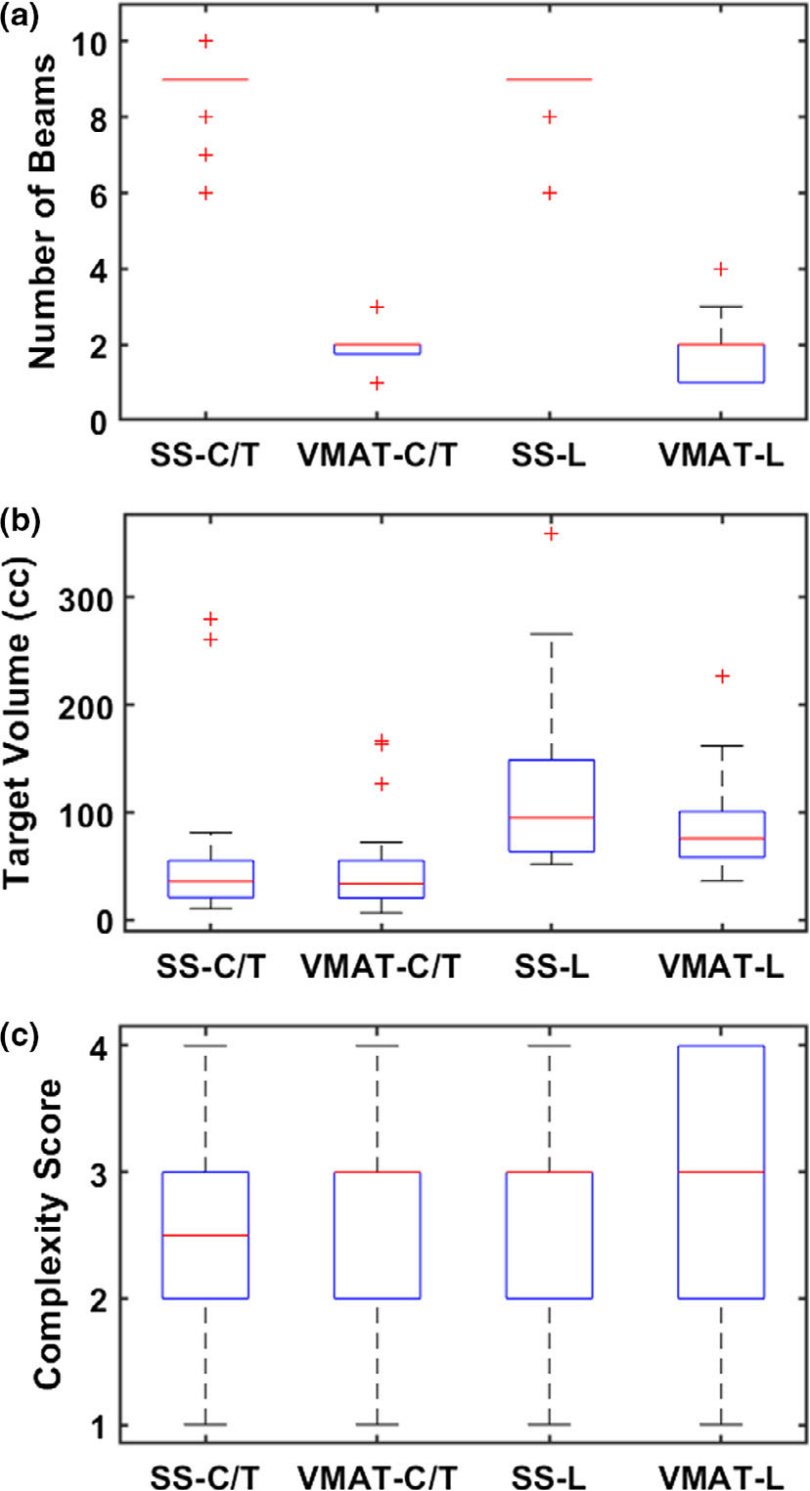
Clinical plan statistics: (a) number of beams, (b) target volumes, and (c) complexity scores. Results are depicted with box plots with median and interquartile range values. Outliers are marked in red crosses (“+”).

As shown in Fig. 3, the ssIMRT plans used a median of nine beams, and the VMAT plans used a median of two arcs. The target volumes between the ssIMRT and VMAT groups were not significantly different: median 35.97 vs 33.8 cc (*P* = 0.897) for C/T spine and median 95.16 vs 75.88 cc (*P* = 0.171) for L spine. The complexity scores were also comparable between the ssIMRT and VMAT groups: median 2.5 vs 3 (*P* = 0.741) for C/T spine and median 3 vs 3 (*P* = 0.187) for L spine.

Tables 4 and 5 summarize the median and range of each dosimetric quantity for ssIMRT and VMAT. As shown in Table 3, using VMAT to treat C/T spine achieved significantly lower V_10Gy_ (4.15% vs 1.85%, *P* = 0.004) and D_0.03cc_ (10.85 vs 10.10 Gy, *P* = 0.032) for the spinal cord compared to ssIMRT. VMAT plans were also more conformal, while both modalities had similar homogeneity. Plan delivery accuracy, in terms of gPR, were comparable for ssIMRT and VMAT plans for C/T spine (*P* = 0.719). On the other hand, there were no significant differences between ssIMRT and VMAT for any of the dosimetric endpoints, conformity, homogeneity, or delivery accuracy for L spine treatment plans (Table 5).

**Table 4:** Dosimetric endpoints and delivery accuracy comparison between step‐and‐shoot intensity modulated radiotheraphy (SSIMRT) and volumetric modulated arc theraphy (VMAT) for C/T spine treatment plans.

	ssIMRT (N = 34)		VMAT (N = 29)		*P*
	Median	Range	Median	Range	
V_10Gy_ (%)	4.15	0.13–7.03	1.85	0–8.46	0.004
D_0.03cc_ (Gy)	10.85	9.60–12.00	10.10	6.90–12.60	0.032
CI	1.280	0.933–1.833	1.080	0.973–1.467	0.009
HI	1.340	1.233–1.578	1.330	1.217–1.539	0.697
gPR (%)	99.10	91.54–99.89	99.17	97.19–99.17	0.719

**Table 5:** Dosimetric endpoints and delivery accuracy comparison between step‐and‐shoot intensity modulated radiotheraphy (SSIMRT) and volumetric modulated arc theraphy (VMAT) for L spine treatment plans.

	ssIMRT (N = 19)		VMAT (N = 16)		*P*
	Median	Range	Median	Range	
V_12Gy_ (%)	0.52	0–7.56	1.65	0–4.68	0.453
D_0.03cc_ (Gy)	12.60	9.70–14.70	13.30	10.70–15.20	0.180
CI	1.050	0.970–1.587	1.020	0.906–1.524	0.826
HI	1.330	1.189–1.433	1.310	1.244–1.578	0.689
gPR (%)	98.03	90.72–99.57	98.34	96.88–99.65	0.276

## 4 DISCUSSION

Compared with clinical ssIMRT plans, spinal cord and cauda equina doses from VMAT replans were reduced in each case as shown with lower V_10Gy_ and D_0.03cc_. Conformality was also improved in plans 1, 2 and 3, which were C/T spine treatments. To investigate the clinical performance of ssIMRT and VMAT planning and delivery methods, we focus on comparison of clinical treatment plans and present population based results, which may overcome the limitations of previous treatment planning and dosimetry comparison studies.^16^ In this study, we included 98 different clinical plans for 74 patients, which is the largest treatment planning study in spine SBRT to the best of our knowledge. Treatment planning in clinical settings is different from those done for research purposes — planners have limited time and no prior knowledge with the exact same anatomy. Furthermore, clinical plans are under careful scrutiny of treating radiation oncologists. The same characteristics apply to IMRT QAs that are done in clinical settings instead of research settings. Therefore, it is important to report the population‐based results so that research and clinical practice are more aligned.

Based on the results, when treating C/T spine with single fraction SBRT, VMAT provided better conformality and better spinal cord sparing compared to ssIMRT. The plan homogeneities of the two modalities were comparable. These findings are similar to some published work^20^, ^21^ but different from others.^14^ The different results could be partly due to the use of different versions of the planning system and the experience of treating single fraction spine SBRT gained over the time. Especially, our study overcame the limitations in comparing clinical plans with research plans. On the other hand, the differences in L spine treatments were not statistically significant. The authors speculate that the insignificance of L spine results are due to the limited sample size. The C/T spine groups have 34 ssIMRT and 29 VMAT plans, while the L spine groups have 19 ssIMRT and 16 VMAT plans.

The five VMAT replans, with the same patient anatomy and dose‐volume constraints, showed comparable EPID QA results to the ssIMRT plans. For the clinical plans that were measured with MatriXX, both ssIMRT and VMAT had median gPR > 98% with the gamma criteria (3%, 3mm). However, the lowest gPR with ssIMRT were 91.54% and 90.72% for C/T and L spine plans, respectively; the lowest with VMAT were 97.19% and 96.88%. Rijken et al.^22^ recently published a study with four patients in spine SBRT QA on an Elekta machine and concluded that increase in plan control points increased the delivery accuracy. In our study, the treatment plans were all delivered on a Varian Edge machine, and the differences in ssIMRT and VMAT QA results did not show statistical significance, although in general the ssIMRT plans had fewer control points compared to the VMAT ones.

In 2009, Wu et al.^13^ published results comparing ssIMRT and VMAT for spine SBRT (16 Gy in one fraction). They found that delivery efficiency was substantially improved with VMAT, but one arc VMAT did not provide as good cord sparing as compared to ssIMRT. At the time of the study, VMAT was a relatively new technology compared to ssIMRT. Over the past decade, VMAT optimizers have been developed in commercial treatment planning systems, clinically tested and significantly improved.^23^, ^24^, ^25^ It is important to note that different planning systems may produce different results. Furthermore, as planners gain clinical and planning experience with VMAT, plan quality also improves significantly.

As reported in a spine SBRT phantom study that compared multiple delivery systems,^16^ CyberKnife (CK) was shown to have the best spinal cord sparing. Using a series of three complex spine lesions, Moustakis et al.^26^ conducted a treatment plan comparison study that involved multiple platforms at different centers. Their conclusion, in contrary, showed that VMAT plans achieved better plan quality than previous established CK radiosurgery benchmarks. Head‐to‐head planning comparisons could be biased because these plans did not undergo careful scrutiny of the treating physicians and carried no clinical impact, which further emphasized the necessity of treatment planning study using clinical plans.

Compared to most of the previous publications in spine SBRT treatment planning and quality assurance,^13^, ^14^, ^20^, ^21^ this study has a significantly larger sample size. Different findings may be partially due to the sample size difference. Note that all plans in this report have a prescription dose of 18 Gy, while other published studies have prescription doses of 14 to 16 Gy. It has become our clinical standard to treat to 18 Gy. Dose constraints to spinal cord, cauda equina, and other OARs have all been met despite the higher prescription dose.

## 5 CONCLUSION

Different from previously published works that were based on research plans using the same patient anatomy, this work was based on our clinically treated plans. We find that the plan quality of VMAT is better than that of ssIMRT for treating cervical and thoracic spine SBRT, achieving adequate target coverage, comparable delivery accuracy, better conformality, and lower dose to the spinal cord. With its improved delivery efficiency, VMAT should be considered a preferred treatment option for single fraction spine SBRT.

## CONFLICT OF INTEREST

None.
